# Loss of RASSF1A Expression in Colorectal Cancer and Its Association with K-ras Status

**DOI:** 10.1155/2013/976765

**Published:** 2013-06-22

**Authors:** Dan Cao, Ye Chen, Yuan Tang, Xing-Chen Peng, Hang Dong, Long-Hao Li, Ke Cheng, Jun Ge, Ji-Yan Liu

**Affiliations:** ^1^Department of Medical Oncology, Cancer Center, The State Key Laboratory of Biotherapy, West China Hospital, West China Medical School, Sichuan University, 37 Guo Xue Xiang, Chengdu, Sichuan 610041, China; ^2^Department of Pathology, West China Hospital, West China Medical School, Sichuan University, Chengdu 610041, China

## Abstract

*Background*. The RAS-association domain family 1 A (RASSF1A) is a classical member of RAS effectors regulating cell proliferation and apoptosis. Loss of RASSF1A expression may shift the balance towards a growth-promoting effect without the necessity of activating K-ras mutations. Its potential association with K-ras mutations in colorectal cancer (CRC) is unclear. *Methods*. RASSF1A expression was examined in normal mucosa, adenoma, and tumor tissues of colon and rectum, respectively. We examined the association of RASSF1A expression, mutations of K-ras, and EGFR status in 76 primary CRCs. The relationship between clinicopathological characteristics and RASSF1A expression was also analyzed. *Results*. RASSF1A expression level decreased progressively in normal mucosa, adenoma and, tumor tissues, and the loss of RASSF1A expression occurred more frequently in tumor tissues. Of 76 primary CRCs, loss of RASSF1A expression and/or K-ras mutations were detected in 77% cases. Loss of RASSF1A expression was more frequent in K-ras wild-type than in mutation cases (63% versus 32%, *P* = 0.011). *Conclusions*. Our study indicates that loss of RASSF1A may be involved in pathogenesis of CRC, its expression was found predominantly in K-ras wild-type CRCs, suggesting that it may be another way of affecting RAS signaling, in addition to K-ras mutations.

## 1. Introduction

Colorectal cancer (CRC) is one of the most commonly diagnosed malignancies worldwide and is still carrying a high morbidity and mortality. At least 50% of CRCs are thought to have a dysregulation of the RAS-RAF-MEK-ERK (also known as the mitogen-activated protein kinase, MAPK) pathway [1, 2]. Among those molecules, K-ras plays an essential role in the initiation of MAPK pathway and has been widely established as an important oncogene since the first report about its mutation [3]. Approximately, one-third of CRCs have been reported to have activating K-ras mutations, which implied insensitivity to EGFR inhibitors. K-ras mutations are found mostly (90%) in codons 12 and 13 but may also affect codon 61 and others [4]. Another mechanism, including mutations of BRAF [5] and NF1 [2], has also been reported to be involved in the overactive RAS signaling pathway.

The RAS-association domain family 1 A (RASSF1A) is a tumor suppressor gene located on chromosome 3p21.3 and is a member of a new group of RAS effectors thought to regulate cell proliferation and apoptosis [6]. RASSF1A has been shown to affect multiple cellular activities, including promotion of apoptosis, cell cycle arrest, and maintenance of genomic stability [7]. It was also reported to suppress the activated K-ras-induced oxidative DNA damage [8]. Mutations in RASSF1A are rare, and methylation is the major mechanism for RASSF1A inactivation. A direct correlation between promoter methylation and loss of RASSF1A expression has been shown in more than half of human cancers [9–12]. It is expressed in all nonmalignant epithelial cells, but not in a large variety of human cancers, including CRC, lung cancers, breast cancers, and ovarian carcinoma [13–19] while, the exogenous expression of RASSF1A decreases colony formation in vitro and tumor formation in vivo [20]. Observations suggest that RASSF1A functions as a tumor suppressor through RAS-mediated apoptosis [6, 7]. All these suggested that it may have a pivotal role in tumor prevention. 

As implied by its designation, RASSF1A is thought to interact with K-ras through a RAS association domain that alters its effects. Loss of RASSF1A expression by methylation may shift the balance towards a growth-promoting effect without the necessity of activating K-ras mutations. Recently, several groups have reported upon the existence of a relationship between RASSF1A and RAS signaling way [20, 21]. Although increasing evidence points to a direct binding between RASSF1A and K-ras, its association to and its effect on K-ras are still not decided [22]. Furthermore, EGFR is expressed in 80% of CRC, and several recent and concordant clinical studies have shown that EGFR status is independent of K-ras mutations in colorectal tumors [23]. However, whether RASSF1A expression is related to K-ras mutation, EGFR status, and clinical feature of CRCs still needs to be clarified.

In this study, in order to explore the role of RASSF1A in colorectal pathogenesis, expression of RASSF1A in normal mucosa, adenoma, and tumor tissues of colon and rectum was studied. Then, its association with clinicopathological characteristics was analyzed in primary CRCs, and its relationship between K-ras mutations and EGFR expression was also investigated.

## 2. Materials and Methods

### 2.1. Sample Collection

Eighty one formalin-fixed, paraffin-embedded tissue samples from patients who underwent surgical resection for CRC during the period between October 2009 and July 2011 in our hospital were obtained. Only 76 samples with records of sufficient tumor tissue and accurate pathological staging were finally included in the study. Twenty of the normal epithelium retrieved up to 5 cm away from the tumor's edge, and 20 of the adenoma from the same patients were also evaluated in the clinical and histological study.

### 2.2. Histology

Fresh CRC samples were received after resection, fixed in 10% pH-neutral formalin, and embedded in paraffin. All the patients had the diagnosis of adenocarcinomas and were staged according to the American Joint Commission for Cancer staging (AJCC/TNM, the sixth version) system. Clinicopathological characteristics in our study included age, gender, tumor size, degree of histological differentiation (well/moderate/poor, WHO), depth of infiltration, and staging. All histological slides were reviewed by two senior pathologists from our institution to confirm the diagnoses and to evaluate the patterns of RASSF1A and EGFR. In the case of differing opinions, the definitive assessment was obtained by consensus.

### 2.3. Immunohistochemistry

Immunohistochemistry was performed using the un-avidin-biotin complex technique named EnVision and MaxVision. Four *μ*m thick sections were cut consecutively from formalin-fixed, paraffin-embedded tissue. Sections were mounted on silanized slides and allowed to dry overnight at 37°C. After deparaffinization and rehydratation, slides were incubated with 3% hydrogen peroxide solution for 5 min. After a washing procedure with the supplied buffer, tissue sections were repaired for 40 min with ethylenediamine tetraacetic acid. The slides were incubated for 60 min at 37°C and then overnight at 4°C with mouse monoclonal anti-RASSF1A (mouse monoclonal, Abcam) and anti-EGFR (mouse monoclonal, Santa Cruz) at a dilution of 1 : 30 and 1 : 300. After three rinses in buffer, the slides were incubated with the secondary antibodies of RASSF1A (Polymerase antibody MaxVision, anti-mouse/rabbit, DakoCytomation) and EGFR (unbiotinylated antibody, EnVisionTM System, HRP, anti-mouse/rabbit, DakoCytomation). Tissue staining was visualized with a DAB substrate chromogen solution (DakoCytomation). Slides were counterstained with hematoxylin, dehydrated, and mounted. Each run included, for each patient, phosphate buffered solution (PBS) used as the primary antibody for the negative controls and normal epithelium known to express RASSF1A and EGFR served as the positive control.

Five fields of vision by high power lens (×400) were selected randomly, and 200 cells were counted per field. Then, the percentage of positive cells was calculated. Nuclear and cytoplasmic reactivity for RASSF1A proteins was considered as positive or negative as described previously [24]. For RASSF1A: −, 0%; +, 1–30%; ++, >30%; % indicates the percentage of the nuclear and cytoplasmic immunostained cells with each individual protein. 

The percentage of labeled cells of EGFR expression was graded as follows: grade 0, no positive cells; grade 1, 1–25% labeled tumor cells; grade 2, 25–50% labeled tumor cells; grade 3, >50% positive tumor cells. The intensity of peroxidase deposits, ranging from light beige to dark brown, was assessed visually as indicating the tumor cell membrane, cytoplasm, or both and was scored as 0 (negative), 1 (weak), 2 (moderate), or 3 (strong). A composite score, potentially ranging from 0 to 9, was obtained by multiplying the grade by the intensity [25]. Patients were analyzed as a function of their EGFR expression: low, <6 and high, ≥6.

### 2.4. DNA Extraction from Paraffin Tissue Blocks

After identification of at least 75% tumor area by a pathologist, tumor tissue was manually dissected from five consecutive 10 *μ*m sections of the paraffin-embedded tissue. The extracted tumor cells were dissolved in a total volume of 190 *μ*L digestion buffer (DNA tissue mini kit, Qiagen) and were treated with proteinase K overnight at 56°C. DNA purification was achieved using a nucleic acid robot device (BIO 101, Qiagen). 

### 2.5. Detection of K-ras Codons 12 and 13 Mutations by Automatic Sequencing

PCR amplification was done in a total volume of 20 *μ*L containing 20 ng genomic DNA, 0.2 mmol/L deoxynucleotide triphosphate, 0.5 units of Taq polymerase (HotStar Taq, Qiagen). The primer sets for codons 12 and 13 of the K-ras gene were 5′-AGGCCTGCTGAAAATGACTGAA-3′ (sense) and 5′-AAAGAATGGTCCTGC ACCAG-3′ (antisense) flanking codons 12 and 13. For DNA sequencing, PCR was performed in a total volume of 10 *μ*L containing the purified PCR products (20–50 ng), 1.6 pmol primer, 1 *μ*L of BigDye terminator Mix, 1x adding buffer, and 0.1 units of Taq Polymerase. Amplification was carried out using one standard and one biotinylated primer. DNA isolated from the CRC cell line was used as positive control. In the negative controls, no DNA was added. Cycle sequencing analysis of PCR fragments was done with the BigDye Terminator system (PE Biosystems) using amplification primers for bidirectional sequencing. The reaction products were analyzed on an ABI PRISM 3700 sequencer (PE Biosystems).

### 2.6. Statistical Analysis

Statistical analyses were all performed using SPSS 13.0 software. To test for difference of RASSF1A expression among normal epithelium, adenoma, and cancer of colorectum, the chi-square analysis was performed for categorical variables. Associations between RASSF1A expression, K-ras mutation, EGFR status, and the clinicopathological parameters and the relationships among these three factors were analyzed using the Fisher's exact test (or chi-square test) for categorical variables.

## 3. Results

### 3.1. RASSF1A Expression in Normal Mucosa, Adenoma, and Colorectal Cancer

**Figure 1:**
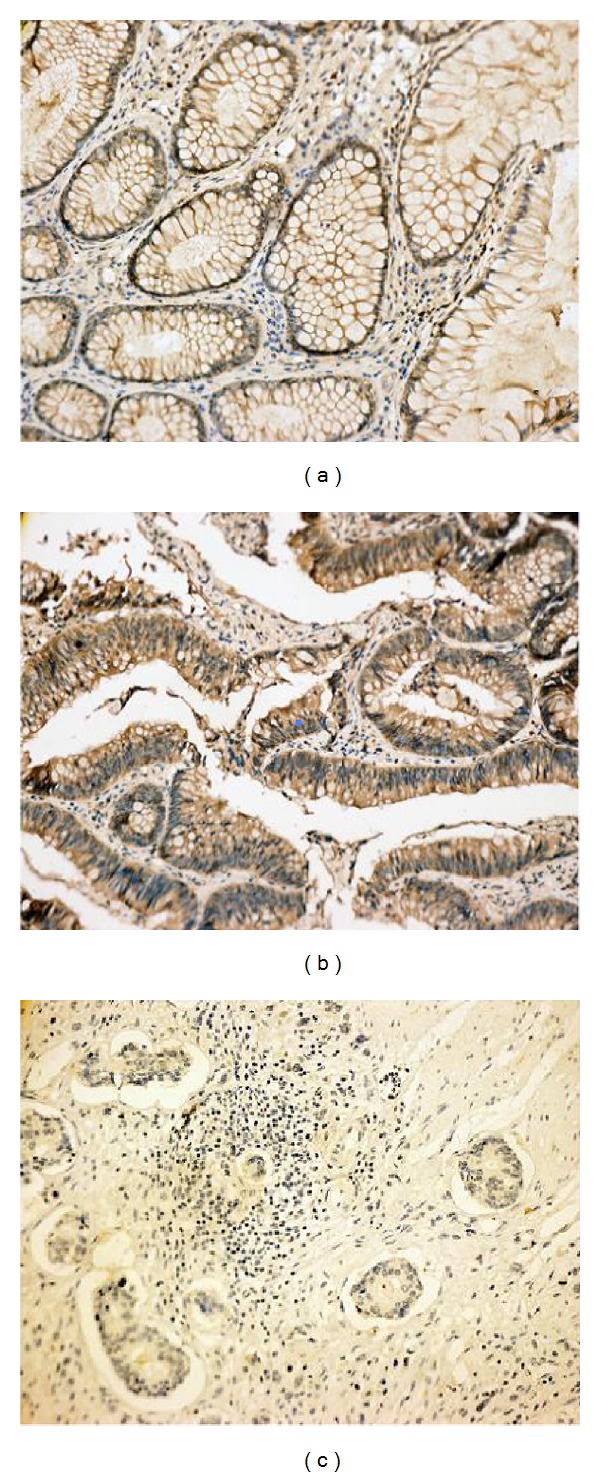
Immunohistochemical staining of RASSF1A in the normal tissue, adenoma, and tumor tissue. (a) Positive expression of RASSF1A in colonic epithelium (MaxVision, ×200). (b) Typical mixed nuclear/cytoplasmic immunostaining of RASSF1A in adenoma (MaxVision, ×200). (c) Negative expression of RASSF1A in colonic (or rectal) carcinoma (MaxVision, ×200).

In this study, RASSF1A expression was detected in normal mucosa, adenoma, and tumor tissues of CRC patients. No patients had history of chemotherapy or radiotherapy before surgery. The pattern of RASSF1A protein expression was mixed nuclear/cytoplasmic staining. The positive expression of RASSF1A was found to be 95% (19/20) in the normal mucosa, 70% (14/20) in the adenoma, and 48.68% (37/76) in the tumor tissues, respectively. RASSF1A expression decreased progressively in the three groups, and the difference was significant (*P* < 0.001Table 1). The loss of RASSF1A protein expression (51.32%) was found to be more frequent in tumor tissues compared to the other two groups (Figure 1).

### 3.2. RASSF1A Expression and Patient Characteristics

In 76 CRC patients, forty-four were male, and the median age was 56 ± 11.5 (30–82). Of the 76 tumor tissues obtained, 38 cases (50%) were located in the colon and 38 cases (50%) in the rectum. Forty-four cases (58%) were well and moderately differentiated adenocarcinoma. Invaded depth of the majority (77%) was T3 and T4. Of the all patients at the time of diagnosis, 20 had distant metastases including radical resectable liver metastases and palliative resectable lung metastases. Negative expression of RASSF1A occurred in 27 of 44 men (61%) and in 12 of 32 women (37%), and the difference was statistically significant (*P* = 0.040). In addition, loss of RASSF1A expression occurred more frequently in carcinoma of colon (24 of 38, 63%) than in carcinoma of rectum (15 of 38, 39%) (*P* = 0.001, Table 2). The difference had no significance between RASSF1A expression and other clinical parameters such as age, tumor size, degree of histological differentiation, depth of infiltration, and stage (*P* > 0.05).

### 3.3.  K-ras Mutation, EGFR Status, and Patient Clinicopathological Features

**Figure 2:**
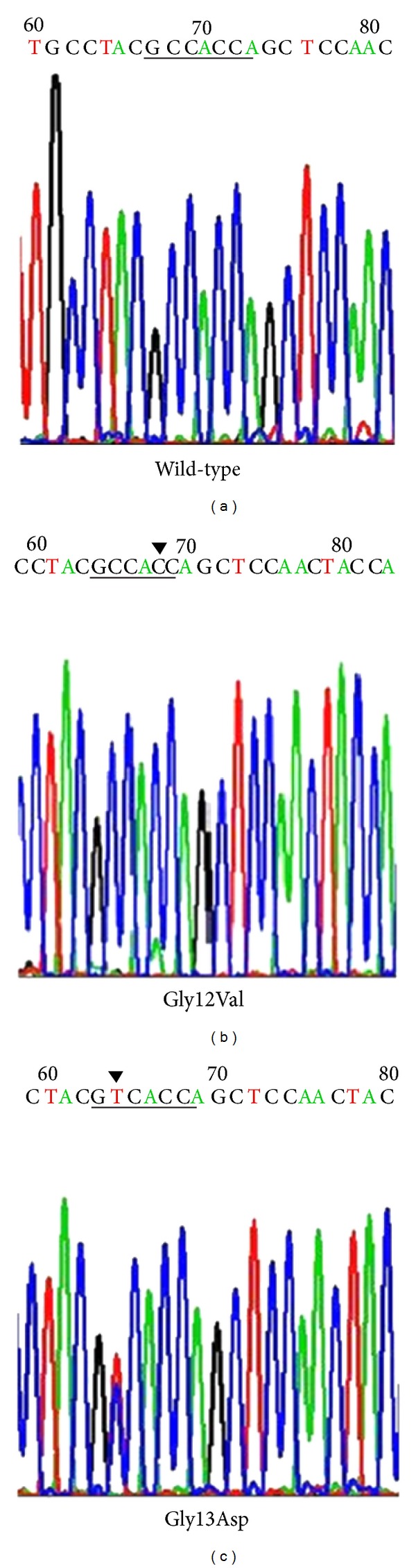
K-ras genotype in CRC. (a) K-ras wild-type. (b) Representative example of K-ras mutation of codon 12. (c) Representative results of K-ras mutation of codon 13.

Twenty-eight (36%) of the 76 CRC samples examined showed a mutation at either codons 12 or 13 of the K-ras gene. Of that 28, 26 (92%) were at codon 12 and 3 (8%) at codon 13; GGT-GTT Gly12Val, GGT-GAT Gly12Asp, and GGC-GAC Gly13Asp were detected in this study (Figure 2). The difference of K-ras mutation had no significance in different age, sex, tumor size, degree of histological differentiation, and stage (*P* > 0.05), but K-ras mutation was significantly associated with depth of infiltration (*P* = 0.015). Mutation rate appeared to be higher in T3/T4 (26 of 59, 44%) than in T1/T2 (2 of 17, 12%).

**Figure 3:**
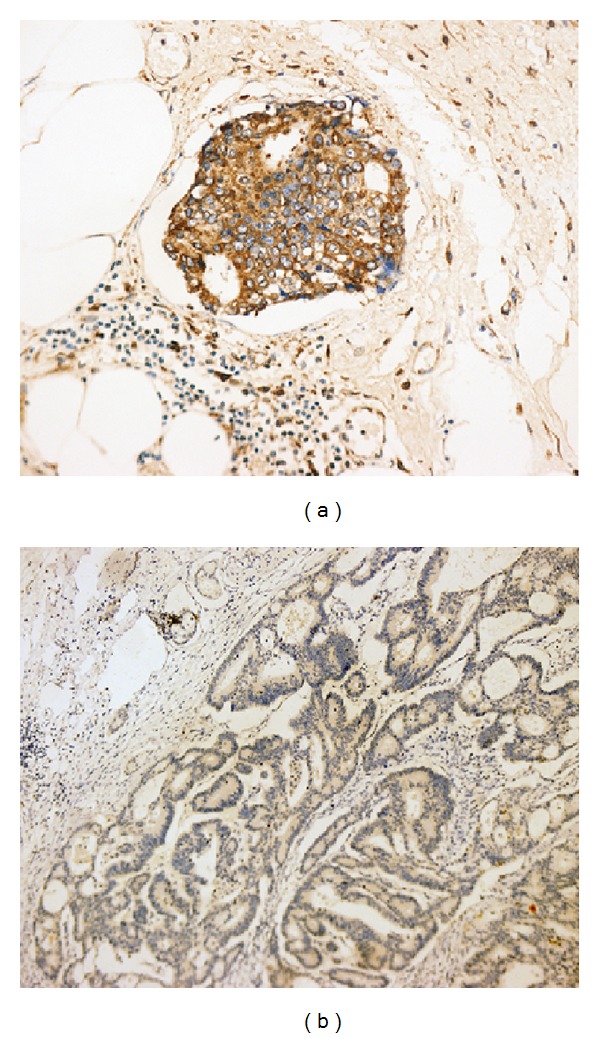
Expression of EGFR in CRC samples. (a) Typical immunoreactivity of membrane and cytoplasm of EGFR in CRC cells (EnVision, ×240). (b) Negative expression of EGFR in CRC tissues (EnVision, ×100).

According to the labeling-intensity scores, EGFR expression was considered high (≥6) and low (<6). High expression of EGFR was found to be 0% (0/20) in the normal mucosa, 5% (1/20) in the adenoma, and 18% (14/76) in the tumor tissues, respectively (Figure 3). The percentage of high EGFR expression increased progressively in the three groups, and the difference was significant (*P* < 0.05). There was no significant association between high expression of EGFR and such clinicopathological factors as age, gender, site, tumor size, degree of histological differentiation, and depth of infiltration (*P* > 0.05). But EGFR overexpression was associated with tumor stage, with the percentage of patients with EGFR overexpression was higher in TNM stage IV than in stages I/II/III CRCs (33% versus 12% respectively, *P* = 0.023) (data not shown).

### 3.4. Association between Loss of RASSF1A Expression, K-ras Mutation, and EGFR Status

Overall, K-ras mutations were observed in 28 of 76 (37%) and loss of RASSF1A was observed in 39 of 76 (51%) cases. Of the 76 patients examined, loss of RASSF1A expression was found to have higher incidence in cases with K-ras wild-type (30 of 48, 63%) than in K-ras mutation (9 of 28, 32%) (*P* = 0.011). 58 of 76 (77%) patients were observed to have loss of RASSF1A expression and/or K-ras mutations. For the 76 adenocarcinomas studied, 18 (23%) had neither K-ras mutation nor loss of RASSF1A expression, and 9 (12%) had both K-ras mutation and loss of RASSF1A expression. Neither the association between RASSF1A expression and EGFR status nor K-ras mutation and EGFR expression had significant difference (*P* = 0.895, Table 3).

## 4. Discussion 

In this study, we investigated expression of RASSF1A, K-ras mutation, and EGFR expression and analyzed the relationships between them in primary CRC in an attempt to understand the role of RASSF1A  in RAS-mediated oncogenic transformation. The relationships between these factors and patients' clinicopathological characteristics were also analyzed.

In accordance with the major studies published to date [26, 27], our results showed that the incidence of positive RASSF1A expression decreased progressively in the normal mucosa, adenoma, and tumor tissues. The loss of RASSF1A protein expression was found to be more obvious in tumor tissues than in the nontumor tissues. High incidence of negative RASSF1A expression in carcinomas and an increased frequency in adenoma indicate that this may be an early event in colorectal carcinogenesis. As we know, the Ras signaling pathway is an essential mediator in the signaling that occurs in cells undergoing CRC, which ultimately results in loss of cell-cell contacts, cytoskeletal remodeling, and increased mobility [28]. On the basis of many observations suggesting that RASSF1A mediates RAS-dependent apoptosis, it was hypothesized that RASSF1A inactivation is closely related to RAS activation in human cancers and thus contributes to malignant transformation by inhibiting RAS-mediated apoptosis [29]. Loss of RASSF1A expression could shift the balance towards a growth-promoting effect as a result of the loss of the proapoptotic and cell cycle-suppressive actions, without the necessity of RAS-activating mutations. Our results showed that the majority of the patients with colorectal cancers was observed to be with loss of RASSF1A expression and/or K-ras mutations. Among them, only a few of patients had both K-ras mutation and loss of RASSF1A expression. Unlike studies in lung cancer [20], in our data, significant potential association was found between loss of RASSF1A expression and K-ras mutation in 76 primary CRCs. The prevalence of the loss of RASSF1A expression in cases with the wild-type K-ras was higher than that in those with the mutant K-ras, and this difference was statistically significant, but the concrete mechanism was not clear. The CRC could be caused by combinatorial effects of various factors including gene mutations and environmental risk factors. K-ras mutations are one of the commonly believed mechanisms of CRC development. Otherwise, the loss of RASSF1A may act together with other risk factors to cause CRC without K-ras mutation. Without the loss of RASSF1A, these risk factors may not be sufficient to cause CRC, which may be the possible mechanism that the loss of RASSF1A is more frequent in K-ras wild-type CRCs. As we know, several data has shown that K-ras mutation plays an important role in activating the RAS pathway in CRC. However, the exact mechanism of RASSF1A functioning as a RAS effector is not well elucidated. According to our data, we speculated that loss of RASSF1A expression might be a complementary mechanism in the onset of colorectal cancer in addition to K-ras mutations. Some researchers had found inactivation of RASSF1A and its synergistic effect with activated K-ras in nasopharyngeal carcinoma [12]. In our study, 11.8% (9/76) of the samples were also found to be both loss of RASSF1A expression and K-ras mutations in CRC; it needs further studies to verify whether inactivation of RASSF1A has synergistic effect with activated K-ras in CRC.

In our study, we also found that frequency of loss of RASSF1A expression appeared to be higher in men compared with women and in carcinoma of colon than in rectum. The significance and the reason were not clear and still need further research and large size observations to clarify. In this series of 76 CRC patients, 36% of the malignant tumors were with K-ras mutated at either codons 12 or 13. Of the mutated K-ras genes in these patients, 92% were mutated at codon 12, 8% at codon 13, and none of them was mutated at both codons 12 and 13. It was interesting to find that K-ras mutation rate appeared to be higher in T3/T4 than in T1/T2, and it probably indicated tumors with the tendency of invasion and metastasis. EGFR is overexpressed in many types of cancers, especially CRC, and the overexpression seems to reflect more aggressive histological and clinical behaviors. EGFR has been found to be elevated in CRCs, with expression rates ranging from 25 to 77% [30]. Our observations confirm that the rate of EGFR overexpression increased progressively from normal mucosa, adenoma to tumor tissues. In addition, we found that EGFR overexpression was associated with tumor stage,  as the percentage of patients with EGFR overexpression was higher in TNM stage IV than in stages I/II/III CRCs. This possibly implied that patients with EGFR overexpression in advanced stage might have poor prognosis. In addition, we found that neither the association between RASSF1A expression and EGFR status nor K-ras mutation and EGFR expression had significant difference. Although another study had showed the inverse correlation of RASSF1A and EGFR in lung cancer [31], the similar phenomenon was not observed in CRC. It is possible that the discrepancy may stem from different tumor types or our limited samples. Further studies will be needed to address the questions.

In conclusion, the high frequency of loss of RASSF1A expression in carcinomas and an increased frequency in adenoma compared to normal tissue indicated that loss of RASSF1A expression might be an early event in CRC carcinogenesis. Importantly, the majority of the patients with colorectal cancers were observed to have K-ras mutations or/and loss of RASSF1A  expression, and loss of RASSF1A expression was more frequently seen in K-ras wild-type cases. Thus, our results suggested that the loss of RASSF1A expression might be a complementary mechanism in the onset of colorectal cancer in addition to K-ras mutations. 

## Figures and Tables

**Table 1:** RASSF1A expression in normal tissue, adenoma, and tumor.

	RASSF1A		χ^2^	*P*
	Positive	Negative		
	No. of cases (%)			
Normal tissue	19 (95%)	1 (5%)	15.135	**0.001**
Adenoma	14 (70%)	6 (30%)		
Tumor	37 (49%)	39 (51%)		

Bold values represent *P* values which are considered to be statistically significant at <0.05.

**Table 2:** Association between loss of RASSF1A expression and clinicopathological factors.

Factors	Total cases	Loss of RASSF1A expression		
	No.	No. (% of total)	χ^2^	*P *
Age				
<60	41	22 (53.66%)	0.196	0.658
≥60	35	17 (48.57%)		
Sex				
Male	44	27 (61.36%)	4.223	**0.040**
Female	32	12 (37.5%)		
Site				
Colon	38	24 (63.16%)	4.266	**0.039**
Rectum	38	15 (39.47%)		
Tumor size				
<5 cm	55	31 (56.36%)	2.030	0.154
≥5 cm	21	8 (38.10%)		
Differentiation grade				
Poor	32	13 (40.63%)	2.529	0.112
Moderate-well	44	26 (59.09%)		
Invasion depth				
T1 + T2	17	11 (64.71%)	1.572	0.210
T3 + T4	59	28 (47.58%)		
Tumor stage				
I/II/III	56	28 (50%)	0.114	0.736
IV	20	11 (55%)		

Bold values represent *P* values which are considered to be statistically significant at <0.05.

**Table 3:** Relationship between loss of RASSF1A expression, K-ras mutation, and EGFR status in CRC.

	RASSF1A expression		χ^2^	*P*
	Negative	Positive		
K-ras status				
Wild-type	30 (40%)	18 (24%)		
Mutated	9 (11%)	19 (25%)	6.523	**0.011**
EGFR expression				
Low	30 (39%)	32 (42%)		
High	9 (12%)	5 (7%)	0.018	0.895

	K-ras status			
	Wild-type	Mutated		

EGFR expression				
Low	40 (52%)	22 (29%)		
High	8 (11%)	6 (8%)	0.267	0.605

Bold values represent *P* values which are considered to be statistically significant at <0.05.
